# Factors contributing to chronic ankle instability: a protocol for a systematic review of systematic reviews

**DOI:** 10.1186/s13643-016-0275-8

**Published:** 2016-06-07

**Authors:** Cassandra Thompson, Siobhan Schabrun, Rick Romero, Andrea Bialocerkowski, Paul Marshall

**Affiliations:** School of Science and Health, Western Sydney University, Campbelltown, Australia; School of Allied Health Sciences, Griffith University, Nathan, Australia

**Keywords:** Systematic review of systematic reviews, Protocol, Chronic ankle instability

## Abstract

**Background:**

Ankle sprains are a significant clinical problem. Researchers have identified a multitude of factors contributing to the presence of recurrent ankle sprains including deficits in balance, postural control, kinematics, muscle activity, strength, range of motion, ligament laxity and bone/joint characteristics. Unfortunately, the literature examining the presence of these factors in chronic ankle instability (CAI) is conflicting. As a result, researchers have attempted to integrate this evidence using systematic reviews to reach conclusions; however, readers are now faced with an increasing number of systematic review findings that are also conflicting. The overall aim of this review is to critically appraise the methodological quality of previous systematic reviews and pool this evidence to identify contributing factors to CAI.

**Methods:**

A systematic review will be conducted on systematic reviews that investigate the presence of various deficits identified in CAI. Databases will be searched using pre-determined search terms. Reviews will then be assessed for inclusion based on the set eligibility criteria. Two independent reviewers will assess the articles for inclusion before evaluating the methodological quality and presence of bias of the included studies; any disagreements will be resolved by discussion between reviewers to reach consensus or by a third reviewer. Data concerning the specific research question, search strategy, inclusion/exclusion criteria, population, method and outcomes will be extracted. Findings will be analysed with respect to the methodological quality of the included reviews.

**Discussion:**

It is expected that this review will clarify the cause of contradicting findings in the literature and facilitate future research directions.

**Systematic review registration:**

PROSPERO CRD42016032592.

**Electronic supplementary material:**

The online version of this article (doi:10.1186/s13643-016-0275-8) contains supplementary material, which is available to authorized users.

## Background

Ankle sprains account for the most common ankle injury in the majority of sporting activities [1]. Despite lateral ankle sprains being considered a trivial injury, prolonged ankle instability and recurrent injury are thought to exceed 70 % in the sporting context [2]. The presence of prolonged (chronic) ankle instability may be characterised by feelings of instability and “giving way” during normal activities of daily living in addition to mechanical laxity [3]. Current understanding of chronic ankle instability (CAI) indicates that although perceived instability, mechanical laxity and recurrent lateral ankle sprains contribute to CAI, these factors may present independently or in combination within an individual [4]. CAI is significant considering the debilitating health and economic consequences such as reduced quality of life [5], time lost from work and the development of early-onset osteoarthritis [6, 7]. Many researchers have examined differences in the control of human movement between persons with CAI and healthy controls in an attempt to better understand the aetiology of injury; however, findings are conflicting.

Since Freeman and colleagues [8] first proposed altered afferent feedback as the primary functional mechanism of recurrent ankle sprains in 1965, researchers have identified altered bone and ligament characteristics [9–12], in addition to kinematic [13–16], neuromuscular [16–18], postural control [19–23], proprioceptive [24–27], strength [28–31] and range of motion deficits [29] in CAI. However, data is inconsistent, with some studies suggesting functional deficits are not present in CAI [32–37]. These discrepancies likely result from methodological differences between studies, which have ultimately confounded the reader’s ability to draw clear conclusions from the literature. To overcome these discrepancies, many researchers have appraised the available evidence in systematic reviews [38–49].

As a result, readers are now faced with a multitude of systematic reviews that also present conflicting findings. While several systematic reviews have suggested that peroneal reaction time is unaltered in CAI [40, 41, 47], two systematic reviews have reported that reaction time is delayed in this population [42, 49]. Furthermore, some reviews have identified no difference in passive or active joint position recognition [40, 41], whereas many other reviews have found that proprioceptive deficits are present in CAI [43, 44, 47, 48]. We hypothesise that differences in the scope and methodological quality of systematic reviews, in addition to subject inclusion criteria and outcome measures of individual studies, will explain inconsistent findings. To address these inconsistencies, it is now necessary to identify, appraise and synthesise the results of published systematic reviews that assess factors contributing to CAI. Thus, this review has three aims: (1) to critically appraise the overall methodological quality of each review, (2) to integrate the available evidence in a meta-analysis and perform sub-analyses based on subject inclusion and measurement of outcomes of the included studies and (3) to formulate a clearer understanding of the deficits associated with CAI and, from these findings, propose likely risk factors for recurrent lateral ankle sprain injuries.

To critically appraise the methodological quality of the systematic reviews, we will assess the risk of bias, validity of the search strategy with respect to the research question, the number of the included primary experimental studies and the homogeneity of the included studies with respect to the defined population, method used and outcomes assessed. Findings concerning the deficits associated with CAI will then be stratified according to the methodological quality of the reviews.

## Methods/design

This protocol was prepared in accordance with the PRISMA-P statement for preferred reporting items for systematic reviews and meta-analyses protocols [50]. A copy of the PRISMA-P statement is included (see Additional file 1).

### Search strategy

A comprehensive search of the literature will be conducted on five databases, including CINAHL, Medline, PubMed, Scopus and Sport Discus. Databases will be searched from inception. The individual search strategy for each database has been included (see Additional file 2).

Keywords related to systematic reviews and chronic ankle instability will be used. The following search strategy will be modified for use in the respective databases. Mesh terms (“ankle”, “ankle joint”, “lateral ligament, ankle”, “cumulative trauma disorders”, “chronic pain”, “ankle injuries”, “sprains and strains”, “joint instability”, “review”, “meta-analysis” and “meta-analysis as topic”) will be used in conjunction with keywords searched in the title and abstract (ankle* OR talocrural, instability OR sprain OR injury, perceived OR chronic OR functional OR mechanical OR recurrent OR repeated OR repetitive, meta-analysis OR systematic).

### Eligibility criteria

#### Population

Our review targets systematic reviews of risk factors associated with CAI in adults and/or children. We define CAI as a multifaceted condition that may present as either mechanical ankle instability (MAI) of lateral ligaments, perceived instability, recurrent ankle sprains or a combination of the three [3, 4]. How each systematic review and the studies within them define chronic ankle instability will be compared to previous classifications [3] endorsed by the International Ankle Consortium, which are outlined in Table 1. How systematic reviews define CAI will be used to assess the homogeneity of the population.

**Table 1:** International ankle consortium classification of CAI participants

Chronic ankle instability inclusion/exclusion criteria	
Inclusion	
	History of at least 1 significant ankle sprain that resulted in inflammation and impaired physical activity. Initial ankle sprain should occur ≥12 months prior to testing. The most recent sprain should be ≥3 months.
	≥2 episodes of “giving way” *and/or* recurrent ankle sprain *and/or* feelings of instability at the ankle that does not result in an ankle sprain.
	Self-reported ankle instability should be confirmed by a validated ankle instability questionnaire (e.g. the Ankle Instability Index, Cumberland Ankle Instability Tool, Identification of Functional Ankle Instability). Degree of instability should be included if relevant to research question (using the Foot and Ankle Ability Measure or Foot and Ankle Outcome Score).
Exclusion	
	History of previous surgeries to musculoskeletal structures including bone, ligaments and/or nerve.
	History of ankle fracture in either lowe limb requiring realignment.
	Acute injury to musculoskeletal structures (sprain, strain or fracture) in the 3 months prior to testing.

#### Outcomes

The review will identify and examine systematic reviews that have examined factors contributing to CAI (i.e. postural control, neuromuscular control, kinematics, kinetics and bone and ligament structure). Deficits may include, but are not limited to, balance, proprioception, functional performance measures, force and joint angle measures (of the hip, knee, ankle and torso), gross muscle activity, spinal and cortical measures of excitability, mechanical laxity and bone alignment in the foot.

#### Study characteristics

Collins and Fauser [51] stated a systematic review is characterised by explicit, transparent and reproducible methods. Systematic reviews should typically include a narrow-focus question, comprehensive literature search, criterion-based evidence selection, strict validity evaluation and an objective/quantitative summary [52]. To be included, systematic reviews need to (i) complete a comprehensive search of the literature with or without meta-analysis, (ii) answer a focused question and (iii) clearly define the criteria of the search strategy and selection and inclusion of studies. Eligible reviews must also be published in a peer-reviewed journal and include adults and/or children with CAI compared to a healthy population. Study selection will not be restricted by language, that is, potential studies published in a language other than English will be translated and assessed for inclusion. Systematic reviews on patients with an injury (fractures, dislocations) or recovering from surgery will be excluded. Studies using a non-systematic review methodology (e.g. RCTs, cohort studies, case-control studies and cross-sectional studies) will be excluded.

### Selection procedure

Two reviewers will screen all articles identified from the search. First, titles of articles returned from initial searches will be screened based on the eligibility criteria outlined above. Second, abstracts identified as potentially relevant based on the title will be assessed using the same criteria. Third, to seriously consider the remaining articles after exclusion based on the abstract, full texts will be screened for applicability. Finally, references of all seriously considered articles will be hand-searched to identify any relevant systematic reviews missed in the search strategy. Any disagreement between the two reviewers over the study relevance will be resolved by discussion to meet a consensus. If consensus is not reached, a third independent reviewer will be asked to assess the review relevance.

### Quality assessment

Systematic review quality and potential bias will be assessed using a modified tool (outlined in Table 2) based on the criteria outlined in the R-AMSTAR tool. R-AMSTAR was originally developed for quality appraisal of the systematic reviews of intervention studies [53]. For appropriate quality assessment of the systematic reviews of observational studies, we modified the R-AMSTAR tool based on the recommended reporting outcomes of the STROBE statement [54] and common biases identified in the observational studies [55]. A copy of the modified R-AMSTAR is also available (see Additional file 3). The PRISMA statement will also be used to screen the methodological reporting of the systematic reviews [56] before critically appraising each review using the modified R-AMSTAR tool [57]. Critical appraisal and data extraction of all relevant studies will be completed by two reviewers. To ensure consistency, findings of the reviewers will be compared. Disagreements between the two reviewers over methodological quality will be resolved through discussion to reach a consensus. If a consensus is not reached by discussion, a third, independent reviewer will then be asked to make a final decision.

**Table 2:** Modified R-AMSTAR

R-AMSTAR item	Criteria	Score
1. Was an “a priori” design provided?	Original R-AMSTAR criteria.	4
2. Was there duplicate study selection and data extraction?	Original R-AMSTAR criteria.	4
3. Was a comprehensive literature search performed?	Original R-AMSTAR criteria.	4
4. Was the status of publication (i.e. grey literature) used as an inclusion criterion?	Original R-AMSTAR criteria.	4
5. Was a list of studies (included and excluded) provided?	Original R-AMSTAR criteria.	4
6. Were the characteristics of the included studies provided?	Original R-AMSTAR criteria.	4
7. Was the scientific quality of the included studies assessed and documented?	Modified to consider methodological quality of observational studies (e.g. recruitment selection, information bias, measurement errors, confounding and other errors).	4
8. Was the scientific quality of the included studies used appropriately in formulating conclusions?	Modified to contain one criterion: The scientific quality is considered in the analysis and the conclusions of the review (e.g. “the results should be interpreted with caution due to poor quality of included studies.”	1
9. Were the methods used to combine the findings of studies appropriate?	Original R-AMSTAR criteria.	4
10. Was the likelihood of publication bias assessed?	Original R-AMSTAR criteria.	4
11. Was the conflict of interest included?	Removed one criterion: An awareness/statement of conflict of interest in the primary inclusion studies. As the included studies are not intervention-based, conflicting interests are also unlikely.	3
Total score		40

Internal validity of the systematic reviews is considered to be compromised by the presence of biases and methodological flaws. The critical appraisal process will involve assessing each review for potential confounding due to missing data (selection bias). Any impact bias and other methodological flaws that may arise on the internal validity of the review will be considered in the synthesis of review findings. Studies will not be excluded based on quality. Instead, evidence synthesis of the systematic reviews will be stratified according to the modified R-AMSTAR and presence of bias.

For the purpose of this review, three items of the R-AMSTAR were modified. R-AMSTAR was developed for systematic reviews of intervention-based studies; thus, relevant “a priori” method considerations for quality-based assessment differ for those of observational studies. For this reason, the considerations for quality assessment based on items of the STROBE statement were used to ensure studies satisfied the “a priori” method item by making them specific for observational studies (e.g. recruitment selection, information bias, measurement errors, confounding and other errors). Furthermore, the authors considered three criteria of item 8 irrelevant for quality assessment of systematic reviews examined in this study as they are not designed to inform clinical recommendations or practice guidelines. Finally, one criterion was removed from item 11, as conflicting interests is not a criterion of the STROBE statement and rarely reported among observational studies.

Using the modified R-AMSTAR, studies will be given a score out of 40. A higher score means potentially higher methodological quality, greater internal validity and lower risk of bias. We will rank the methodological quality of systematic reviews based on a method previously outlined by Kung et al. [53]. Studies with high internal validity and low risk of bias will be separated from studies with a high risk of bias for consideration in qualitative analysis. Corresponding authors will be contacted if additional information is required to complete the quality appraisal.

### Data extraction and synthesis

Articles will be stored and managed using Endnote X7 throughout the review process. Two reviewers will independently extract data from each systematic review and consolidate findings based on methodological quality, to build evidence tables. The extracted data will be compared between reviewers to ensure consistency. The data extracted will include specific details about the research question, search strategy, inclusion/exclusion criteria, population (sample size and participant characteristics), method and outcomes of significance to the review question and specific objectives. The findings/conclusions will be recorded and synthesised including standard mean difference and 95 % confidence intervals of individual studies provided by meta-analyses, if available. This information will be used to assess the homogeneity of the primary studies with respect to their participant selection/inclusion and methods used to measure outcomes. In the case of missing data, the authors will be contacted directly for a maximum of three times. If no response is received from the authors after the third attempt, the data will be reported as unattainable.

### Data analysis

The percentage agreement between reviewers concerning the methodological quality of systematic reviews will be calculated using kappa scores of agreement and 95 % confidence intervals.

A meta-analysis of meta-analyses will be performed on systematic reviews of the same methodological quality if patient population, intervention and outcomes are comparable. To avoid confounding from the same individual papers being analysed by different systematic reviews, averages and standard deviations of individual studies will be used for meta-analysis instead of the summated mean difference calculated for all studies. During this process, any duplicate studies will be removed. If a meta-analysis is able to be performed, heterogeneity will be assessed using the chi-square (*I*^2^) calculation and interpreted as 0–40 % representing unimportant heterogeneity, 41–60 % moderate heterogeneity, 61–90 % substantial heterogeneity and 91–100 % considerable heterogeneity. If data is identified as heterogeneous, a random-effects model will be used during analysis. When statistical pooling is not possible, non-pooled data will be presented and in table form. Sub-group analyses will be performed with respect to inclusion/exclusion criteria, participant characteristics (unilateral/bilateral or functional/mechanical instability), method used to measure outcomes and methodological quality of the systematic review. All meta-analysis will be conducted in RevManager version 5.0 (Copenhagen: the Nordic Cochrane Centre, the Cochrane Collaboration, 2008).

## Discussion

This review will assess the presence of deficits and thus likely factors that contribute to CAI. Consequently, the review will critically appraise the methodological quality of previously published systematic reviews in attempt to clarify the cause of contradicting findings in the literature and improve transparency in the field. It is hoped that the review will facilitate future research directions and potentially identify new mechanisms for more targeted rehabilitation programmes.

## Abbreviations

CAI, chronic ankle instability; MAI, mechanical ankle instability; PRISMA, Preferred Reporting Items for Systematic Review and Meta-Analysis; PRISMA-P, Preferred Reporting Items for Systematic Review and Meta-Analysis Protocols; R-AMSTAR, Revised Assessment of Multiple Systematic Reviews; STROBE, Strengthening the Reporting of Observational Studies in Epidemiology

## Additional files

Additional file 1: PRISMA-P (Preferred Reporting Items for Systematic review and Meta-Analysis Protocols) 2015 checklist: recommended items to address in a systematic review protocol*. Completed PRISMA-P checklist outlining how each point has been addressed in the manuscript. (PDF 162 kb) Additional file 2: Search strategies. Specific search strategy used for the five databases included in the review, comprising of both keywords and Mesh terms. (PDF 74.3 kb) Additional file 3: Modified R-AMSTAR checklist—quality assessment for systematic reviews of observational studies (adapted from R-AMSTAR). Modified R-AMSTAR tool used to appraise the quality of each systematic review. (PDF 195 kb)
