# Supplementary material for: Factors contributing to chronic ankle instability: a protocol for a systematic review of systematic reviews
Source: Syst Rev. 2016 Jun 7;5:94. doi: 10.1186/s13643-016-0275-8 (PMC4897901; doi:10.1186/s13643-016-0275-8)
Supplement: Additional file 1: — PRISMA-P (Preferred Reporting Items for Systematic review and Meta-Analysis Protocols) 2015 checklist: recommended items to address in a systematic review protocol*. Completed PRISMA-P checklist outlining how each point has been addressed in the manuscript. (PDF 162 kb) [file 13643_2016_275_MOESM1_ESM.pdf]

Additional file 1. PRISMA-P (Preferred Reporting Items for Systematic review and Meta-Analysis Protocols) 2015 checklist: recommended items to address in a systematic review protocol*

Section and topic	Item No	Checklist item
**ADMINISTRATIVE INFORMATION**		
Title:		
Identification	1a	Identify the report as a protocol of a systematic review<br>-Yes “Factors contributing to chronic ankle instability: a protocol for a systematic review of systematic reveiws.”
Update	1b	If the protocol is for an update of a previous systematic review, identify as such<br>-N/A
Registration	2	If registered, provide the name of the registry (such as PROSPERO) and registration number<br>-Not yet registered. Will be registered once protocol is peer-reviewed and finalised.
Authors:		
Contact	3a	Provide name, institutional affiliation, e-mail address of all protocol authors; provide physical mailing address of corresponding author<br>-Yes, please see title page
Contributions	3b	Describe contributions of protocol authors and identify the guarantor of the review<br>-Yes, please see section “CONTRIBUTIONS” of manuscript
Amendments	4	If the protocol represents an amendment of a previously completed or published protocol, identify as such and list changes; otherwise, state plan for documenting important protocol amendments<br>-NA
Support:		
Sources	5a	Indicate sources of financial or other support for the review<br>-N/A
Sponsor	5b	Provide name for the review funder and/or sponsor<br>-N/A
Role of sponsor or funder	5c	Describe roles of funder(s), sponsor(s), and/or institution(s), if any, in developing the protocol
**INTRODUCTION**		
Rationale	6	Describe the rationale for the review in the context of what is already known<br>-Yes:<br>“… data is inconsistent, with some studies suggesting functional [biomechanical] deficits are not present in CAI [30-35]… methodological differences between studies, which have ultimately confounded the reader’s ability to draw clear conclusions from the literature. To overcome these discrepancies, many researchers have appraised the available evidence

		in systematic reviews [36-47]. As a result, readers are now faced with a multitude of systematic reviews that also present conflicting findings.”
Objectives	7	Provide an explicit statement of the question(s) the review will address with reference to participants, interventions, comparators, and outcomes (PICO)<br>-Yes:<br>“We hypothesise that differences in the methodological quality of [previous] systematic reviews and scope of included studies are likely to explain inconsistent findings... Thus, this review has two aims: 1) to critically appraise the methodological quality of these systematic reviews to identify why inconsistencies occur in the literature and, 2) to formulate a clearer understanding of the biomechanical characteristics associated with CAI and from these findings, propose likely risk factors for recurrent ankle sprain injuries.”
**METHODS**		
Eligibility criteria	8	Specify the study characteristics (such as PICO, study design, setting, time frame) and report characteristics (such as years considered, language, publication status) to be used as criteria for eligibility for the review<br>-Yes, our eligibility criteria considers population, outcomes assessed and study characteristics.
Information sources	9	Describe all intended information sources (such as electronic databases, contact with study authors, trial registers or other grey literature sources) with planned dates of coverage<br>-Yes, information sources include electronic databases and contact with authors.
Search strategy	10	Present draft of search strategy to be used for at least one electronic database, including planned limits, such that it could be repeated<br>-Yes, pleas also see supplementary material
Study records:		
Data management	11a	Describe the mechanism(s) that will be used to manage records and data throughout the review<br>-Yes, “Articles will be stored and managed using Endnote X7 throughout the review process”
Selection process	11b	State the process that will be used for selecting studies (such as two independent reviewers) through each phase of the review (that is, screening, eligibility and inclusion in meta-analysis)<br>-Yes “Two reviewers will screen all articles identified from the search. First, titles of articles returned from initial searches will be screened based on the eligibility criteria outlined above. Second, abstracts identified as potentially relevant based on title will be assessed using the same criteria. Third, to seriously consider the remaining articles after exclusion based on abstract, full-texts will be screened for applicability. Finally, references of all seriously considered articles will be hand-searched to identify any relevant systematic reviews missed in the search strategy. Any disagreement between the two reviewers over study relevance will be resolved by discussion to meet a consensus. If consensus is not reached, a third independent reviewer will be asked to assess the reviews relevance.”
Data collection process	11c	Describe planned method of extracting data from reports (such as piloting forms, done independently, in duplicate), any

		processes for obtaining and confirming data from investigators<br>-Yes, "The lead author will extract data from each systematic review and consolidate findings based on methodological quality, to build evidence tables. A second reviewer will check the extracted data."
Data items	12	List and define all variables for which data will be sought (such as PICO items, funding sources), any pre-planned data assumptions and simplifications<br>-Yes, "The data extracted will include specific details about the research question, search strategy, inclusion/exclusion criteria, population (sample size and participant characteristics), method and outcomes of significance to the review question and specific objectives. The findings/conclusions will be recorded and synthesised including, standard mean difference and 95% confidence intervals of individual studies provided by meta-analyses, if available."
Outcomes and prioritization	13	List and define all outcomes for which data will be sought, including prioritization of main and additional outcomes, with rationale<br>-Yes
Risk of bias in individual studies	14	Describe anticipated methods for assessing risk of bias of individual studies, including whether this will be done at the outcome or study level, or both; state how this information will be used in data synthesis<br>-The presence of bias assessment on individual studies within the systematic reviews will be considered in the quality analysis.
Data synthesis	15a	Describe criteria under which study data will be quantitatively synthesised<br>-Yes
	15b	If data are appropriate for quantitative synthesis, describe planned summary measures, methods of handling data and methods of combining data from studies, including any planned exploration of consistency (such as I^2, Kendall's τ)<br>-Yes, homogeneity will be assessed using the Chi-square analysis. If data is heterogeneous, a random-effects model will be used.
	15c	Describe any proposed additional analyses (such as sensitivity or subgroup analyses, meta-regression)<br>-Yes, sub-group analyses will be performed on inclusion/exclusion criteria, participant characteristics (unilateral/bilateral or functional/mechanical instability), method used to measure biomechanical characteristics and methodological quality of the systematic review, if data is sufficient.
	15d	If quantitative synthesis is not appropriate, describe the type of summary planned<br>-Yes, data will be presented descriptively in table format.
Meta-bias(es)	16	Specify any planned assessment of meta-bias(es) (such as publication bias across studies, selective reporting within studies)<br>-Yes, whether systematic reviews have assessed the presence of relevant biases will be considered during appraising the quality of the systematic reviews.
Confidence in cumulative evidence	17	Describe how the strength of the body of evidence will be assessed (such as GRADE)<br>-Yes using the modified R-AMSTAR tool attached in supplementary material

*** It is strongly recommended that this checklist be read in conjunction with the PRISMA-P Explanation and Elaboration (cite when available) for important clarification on the items. Amendments to a review protocol should be tracked and dated. The copyright for PRISMA-P (including checklist) is held by the PRISMA-P Group and is distributed under a Creative Commons Attribution Licence 4.0.**

From: Shamseer L, Moher D, Clarke M, Ghersi D, Liberati A, Petticrew M, Shekelle P, Stewart L, PRISMA-P Group. Preferred reporting items for systematic review and meta-analysis protocols (PRISMA-P) 2015: elaboration and explanation. BMJ. 2015 Jan 2;349(jan02 1):g7647.
