# Supplementary material for: Factors contributing to chronic ankle instability: a protocol for a systematic review of systematic reviews
Source: Syst Rev. 2016 Jun 7;5:94. doi: 10.1186/s13643-016-0275-8 (PMC4897901; doi:10.1186/s13643-016-0275-8)
Supplement: Additional file 2: — Search strategies. Specific search strategy used for the five databases included in the review, comprising of both keywords and Mesh terms. (PDF 74.3 kb) [file 13643_2016_275_MOESM2_ESM.pdf]

Additional file 2. Search strategies

CINAHL
((MH "Review") OR (MH "Meta-Analysis") OR (MH "Meta-Analysis as Topic") OR systematic review OR meta-analysis OR meta analysis) AND ((MH "Ankle") OR (MH "Ankle Joint") OR (MH "Lateral Ligament, Ankle") OR ankle* OR talocrural OR talo-crural OR talo-calcaneal) AND ((MH "Ankle Injuries") OR (MH "Sprains and Strains") OR (MH "Joint Instability") OR sprain OR injur* OR instability*) AND ((MH "Cumulative Trauma Disorders") OR (MH "Chronic Pain") OR perceived OR repetitive OR functional OR mechanical OR recurrent OR repeated OR chronic)

PubMed
((((((((((Cumulative trauma disorders[MeSH Terms]) OR chronic pain[MeSH Terms]) OR repetitive[Title/Abstract]) OR functional[Title/Abstract]) OR mechanical[Title/Abstract]) OR recurrent[Title/Abstract]) OR repeated[Title/Abstract]) OR chronic*[Title/Abstract])) AND ((((((ankle injuries[MeSH Terms]) OR (sprains and strains[MeSH Terms])) OR joint instability[MeSH Terms]) OR sprain[Title/Abstract]) OR injur*[Title/Abstract]) OR instability*[Title/Abstract])) AND ((((((meta-analysis[MeSH Terms]) OR meta-analysis as topic[MeSH Terms]) OR systematic review[Title/Abstract]) OR meta-analysis[Title/Abstract]) OR meta analysis[Title/Abstract]))) AND (((((ankle[MeSH Terms]) OR ankle joint[MeSH Terms]) OR lateral ligament, ankle[MeSH Terms]) OR ankle[Title/Abstract]) OR talocrural OR talo-crural OR talo-calcaneal[Title/Abstract])

MEDLINE
((MH "Review") OR (MH "Meta-Analysis") OR (MH "Meta-Analysis as Topic") OR systematic review OR meta-analysis OR meta analysis) AND ((MH "Ankle") OR (MH "Ankle Joint") OR (MH "Lateral Ligament, Ankle") OR ankle* OR talocrural OR talo-crural OR talo-calcaneal) AND ((MH "Ankle Injuries") OR (MH "Sprains and Strains") OR (MH "Joint Instability") OR sprain OR injur* OR instability*) AND ((MH "Cumulative Trauma Disorders") OR (MH "Chronic Pain") OR perceived OR repetitive OR functional OR mechanical OR recurrent OR repeated OR chronic)

Scopus
INDEXTERMS(Review OR Meta-Analysis OR Meta-Analysis as Topic) OR TITLE-ABS-KEY(systematic review OR meta-analysis OR meta analysis)] AND INDEXTERMS(Ankle OR Ankle Joint OR Lateral Ligament, Ankle) OR TITLE-ABS-Key(ankle* OR talocrural OR talo-crural OR talocalcaneal)] AND INDEXTERMS(Ankle Injuries OR Sprains and Strains OR Joint Instability) OR TITLE-ABS-KEY(sprain OR injur* OR instability*)] AND INDEXTERMS(Cumulative Trauma Disorders OR Chronic Pain) OR TITLE-ABS-KEY(perceive OR repetitive OR functional OR mechanical OR recurrent OR repeated OR chronic)]

SportDiscus

(("Review"[Mesh] OR "Meta-Analysis"[Mesh] OR "Meta-Analysis as Topic"[Mesh] OR systematic review OR meta-analysis OR meta analysis) AND ("Ankle"[Mesh] OR "Ankle Joint"[Mesh] OR “Lateral Ligament, Ankle”[Mesh] OR ankle* OR talocrural OR talo-crural OR talocalcaneal) AND (“Ankle Injuries”[Mesh] OR “Sprains and Strains”[Mesh] OR “Joint Instability”[Mesh] OR sprain OR injur* OR instability*) AND (“Cumulative Trauma Disorders”[Mesh] OR “Chronic Pain”[Mesh] OR perceived OR repetitive OR functional OR mechanical OR recurrent OR repeated OR chronic)
