# Supplementary material for: Factors contributing to chronic ankle instability: a protocol for a systematic review of systematic reviews
Source: Syst Rev. 2016 Jun 7;5:94. doi: 10.1186/s13643-016-0275-8 (PMC4897901; doi:10.1186/s13643-016-0275-8)
Supplement: Additional file 3: — Modified R-AMSTAR checklist—quality assessment for systematic reviews of observational studies (adapted from R-AMSTAR). Modified R-AMSTAR tool used to appraise the quality of each systematic review. (PDF 195 kb) [file 13643_2016_275_MOESM3_ESM.pdf]

Additional file 3: modified R-AMSTAR checklist - quality assessment for Systematic Reviews of observational studies (adapted from R-AMSTAR)

Amstar Item	Criteria
**1. Was an ‘‘a priori’’ design provided?**<br>The research question and inclusion criteria should be established before the conduct of the review.	A A clearly focused (PICO-based) question<br>B Description of inclusion criteria<br>C Study protocol is published and/or registered in advance
**2. Was there duplicate study selection and data extraction?**<br>There should be at least two persons who independently extracted data and a consensus procedure for disagreements should be in place.	A At least two persons independently extracted the data, explicitly stated<br>B Statement of consensus procedure for disagreements<br>C Disagreements among extractors resolved properly as stated or implied
**3. Was a comprehensive literature search performed?**<br>At least two electronic sources should be searched. The report must include years and databases used (e.g., Pubmed, Scopus and MEDLINE). Key words and/or MESH terms must be stated and where feasible the search strategy should be provided. All searches should be supplemented by consulting current contents, reviews, textbooks, specialized registers, or experts in the particular field of study, and by reviewing the references in the studies found.	A At least two electronic sources are searched<br>B Years and databases used are mentioned<br>C Key words and/or MESH terms are stated and where feasible the search strategy outline is provided<br>D Searches should are supplemented by consulting current contents, reviews, textbooks, registers and by reviewing the references in the studies found<br>E Journals are hand-searched or manually searched
**4. Was the status of publication (i.e. grey literature) used as an inclusion criterion?**<br>The authors should state that they searched for reports regardless of their publication type. The authors should state whether or not they excluded any reports (from the systematic review), based on their publication status, language etc.<br>*Note: If review indicates that there was a search for “grey literature” or “unpublished literature,” indicate “yes.” SIGLE database, dissertations, conference proceedings, and trial registries are all considered grey for this purpose. If searching a source that contains both grey and non-grey, must specify that they were searching for grey/unpublished lit.*	A The authors state that they searched for reports regardless of their publication type.<br>B The authors state whether or not they excluded any reports based on their publication status, language etc.<br>C “Non-English papers were translated” or readers sufficiently trained in foreign language<br>D No language restriction or recognition of non-English articles
**5. Was a list of studies (included and excluded) provided?**<br>A list of included and excluded studies should be provided.<br>Explanation: “Excluded studies” refers to those studies seriously considered on the basis of title and/or abstract, but rejected after reading the body of the text.	A Table/list/figure of **included** studies, a reference list does not suffice<br>B Table/list/figure of **excluded** studies either in the article or in a supplemental source<br>C Satisfactory/sufficient statement of the reason for exclusion of the seriously considered studies<br>D Reader is able to retrace the included and the excluded studies anywhere in the article bibliography, reference or supplemental source

**6. Were the characteristics of the included studies provided?**<br>In an aggregated form, such as a table, data from the original studies should be provided on the participants, interventions/exposure, and outcomes.<br>The ranges of characteristics in all the studies analyzed, e.g., age, race, sex, relevant socioeconomic data, disease status, duration, severity, or other diseases should be reported.	A In an aggregated form such as a table, data from the original studies are provided on the participants, interventions/exposure and outcomes<br>B Ranges are provided of the **relevant** characteristics in the studies analyzed<br>C The information provided appears to be complete and accurate
**7. Was the scientific quality of the included studies assessed and documented?**<br>'A priori' methods of assessment should be provided (identified selection mechanisms in recruitment, information bias, measurement errors, confounding and other errors).<br>*Note: Can include use of a quality scoring tool or checklist, e.g., risk of bias, sensitivity analysis, etc., or a description of quality items, with some kind of result for EACH study ("low" or "high" is fine, as long as it is clear which studies scored "low" and which scored "high"; a summary score/range for all studies is not acceptable).*	A 'A priori' methods are provided<br>B The scientific quality of the included studies appears to be meaningful<br>C Discussion/recognition/awareness of level of evidence is present<br>D Quality of evidence is rated/ranked base on characterized instruments
**8. Was the scientific quality of the included studies used appropriately in formulating conclusions?**<br>The results of the methodological rigor and scientific quality should be considered in the analysis and the conclusions of the review, and explicitly stated in formulating recommendations.	A The results of the methodological rigor and scientific quality should be considered in the analysis and the conclusions of the review.<br>*Note: Might say something such as "the results should be interpreted with caution due to poor quality of included studies."*
**9. Were the methods used to combine the findings of studies appropriate?**<br>For the pooled results, a test should be done to ensure the studies were combinable, to assess their homogeneity (i.e., Chi-squared test for homogeneity, I2). If heterogeneity exists, a random effects model should be used and/or the clinical appropriateness of combining should be taken into consideration (i.e., is it sensible to combine?).	A Statement of criteria that were used to decide that the studies analyzed were similar enough to be pooled<br>B For the pooled results, a test is done to ensure the studies were combinable, to assess their homogeneity<br>C a recognition of heterogeneity or lack of thereof is present<br>D If heterogeneity exists a 'random effects model' is used and/or the rationale of combining is taken into consideration<br>E If homogeneity exists, author state a rationale or a statistical test
**10. Was the likelihood of publication bias assessed?**<br>An assessment of publication bias should include a combination of graphical aids (e.g., funnel plot, other available tests) and/or statistical tests (e.g., Egger regression test).	A Recognition of publication bias or file-drawer effect<br>B Graphical aids (e.g. funnel plot)<br>C Statistical tests (e.g. Egger regression test)
**11. Was the conflict of interest included?**<br>Potential sources of support should be clearly acknowledged in both the systematic review and the included studies.	A Statement of sources of support<br>B No conflict of interest. This is subjective and may require some deduction or searching.
